# Gravity sensing in plant and animal cells

**DOI:** 10.1038/s41526-020-00130-8

**Published:** 2021-02-08

**Authors:** Ken Takahashi, Hideyuki Takahashi, Takuya Furuichi, Masatsugu Toyota, Makoto Furutani-Seiki, Takeshi Kobayashi, Haruko Watanabe-Takano, Masahiro Shinohara, Takuro Numaga-Tomita, Asako Sakaue-Sawano, Atsushi Miyawaki, Keiji Naruse

**Affiliations:** 1 Department of Cardiovascular Physiology, Graduate School of Medicine, Dentistry and Pharmaceutical Sciences, Okayama University, Okayama, Japan; 2 Graduate School of Life Sciences, Tohoku University, Sendai, Japan; 3 Faculty of Human Life Sciences, Hagoromo University of International Studies, Sakai, Japan; 4 Department of Biochemistry and Molecular Biology, Saitama University, Saitama, Japan; 5 Department of Systems Biochemistry in Regeneration and Pathology, Graduate School of Medicine, Yamaguchi University, Yamaguchi, Japan; 6 Department of Integrative Physiology, Graduate School of Medicine, Nagoya University, Nagoya, Japan; 7 Department of Cell Biology, National Cerebral and Cardiovascular Center Research Institute, Suita, Osaka Japan; 8 Department of Rehabilitation for the Movement Functions, Research Institute, National Rehabilitation Center for Persons with Disabilities, Tokorozawa, Japan; 9 Department of Molecular Pharmacology, Shinshu University School of Medicine, Matsumoto, Japan; 10 Lab for Cell Function and Dynamics, CBS, RIKEN, Wakō, Saitama Japan

**Keywords:** Cell biology, Molecular biology

## Abstract

Gravity determines shape of body tissue and affects the functions of life, both in plants and animals. The cellular response to gravity is an active process of mechanotransduction. Although plants and animals share some common mechanisms of gravity sensing in spite of their distant phylogenetic origin, each species has its own mechanism to sense and respond to gravity. In this review, we discuss current understanding regarding the mechanisms of cellular gravity sensing in plants and animals. Understanding gravisensing also contributes to life on Earth, e.g., understanding osteoporosis and muscle atrophy. Furthermore, in the current age of Mars exploration, understanding cellular responses to gravity will form the foundation of living in space.

## Introduction

Gravity defines the morphology of life on Earth. It affects the growth and development of plants and animals by regulating the proliferation of their constituent cells^1^. Gravity also plays crucial roles in cellular function. For example, plants grow leaves and roots in the correct direction by sensing gravity^2^. Animals regulate the densities of bones and muscles in response to gravitational load^3,4^. A response to gravity is an active activity inherent to the physiology of plants and animals.

Historically, elucidation of gravity sensing mechanisms in life originates from the study of plants. Abundant research in plant biology has laid the foundation for studying gravity sensing mechanisms in animals. In the first half of this article, we review the mechanisms of gravity sensing in plants from the viewpoint of cellular and molecular biology. Subsequently, the mechanisms of gravity sensing in animal cells will be discussed. Since both lunar base construction and manned Mars exploration plans are being discussed at present^5,6^, discussions regarding bone loss and muscle atrophy in microgravity environments are inevitable. This paper also summarizes the recent findings in this field. Through these discussions, we outline the common mechanisms of gravity sensing in plants and animals.

This paper expands on our understanding of gravity sensing in plant and animal cells and discusses the future direction of gravitational biology, with the ultimate purpose of contributing to the development of living in space.

## Gravity sensing in plants

### Auxin regulation of gravimorphogenesis in plants

**Fig. 1:**
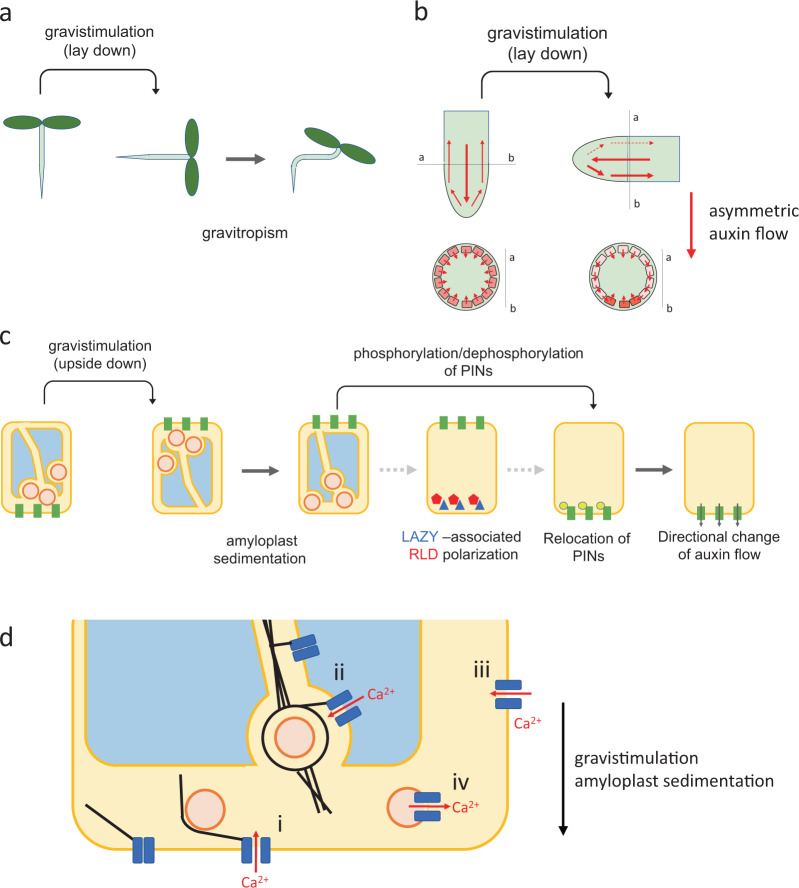
Mechanism for directional growth in response to gravity in plants. **a** Model of gravitropism. **b** Asymmetric auxin flow in horizontally reoriented plants, **c** cellular responses in gravisensing (endodermal) cells. At first, gravity causes sedimentation of amyloplasts. RLD proteins associated with LAZY proteins get polarized to the new bottom side. The LAZY proteins regulate the localization of PIN proteins, which are efflux carriers of plant hormone auxin. Finally, the change in direction of auxin flow causes asymmetric growth of plants. **d** Activation of mechanosensitive ion channels in plasma- and endomembrane upon amyloplast sedimentation (i, ii), deformation, compression and shear stress (iii), displacement of amyloplast (iv).

The survival of sessile organisms, such as plants, depends upon their ability to avoid or mitigate various environmental stresses to which they are subjected. As one of such strategies, plants possess an ability to control directional growth by gravitropism (Fig. 1). Typically, coleoptiles and stems (shoots) grow upward in order to obtain light (negative gravitropism), whereas roots grow downward to acquire water and minerals (positive gravitropism). When plants in a vertical position are reoriented horizontally, the pattern of gravitropic response differs among plant species and organs. Other aspects of plant growth and development are also regulated by gravity. Accordingly, the terms “gravimorphism” or “gravimorphogenesis” can be used for gravity-regulated phenomena in plants.

The plant hormone auxin plays an important role in plant gravimorphogenesis. A major endogenous auxin is indole-3-acetic acid (IAA) that regulates various aspects of plant growth and development. In auxin signaling pathway, Aux/IAA proteins inactivate the auxin response factor (ARF) when auxin level is low^7^. A high level of auxin results in forming a complex of the transcriptional repressor, Aux/IAA, and the AUXIN SIGNALING F-BOX PROTEIN co-repressor/auxin receptor, TIR/AFBs, which allows the degradation of Aux/IAA and the release of ARF repression for modulating the expressions of auxin-related genes^7^. Auxin concentration in the tissues is determined by regulating its biosynthesis, inactivation, and transport. A unique feature of auxin is the polar auxin transport that contributes to most of the directional auxin transport and local auxin concentration. This polar auxin transport is regulated by auxin efflux carriers PIN-FORMED (PIN) family and auxin influx carriers AUX/LAX family proteins. It is considered that auxin is directionally transported across the plasma membrane where PINs localize with a polarity^8,9^. ATP-binding cassette ABC transporters of the B class family (ABCB) also play a role in polar auxin transport^10,11^. TWISTED DWARF1 (TWD1) has been shown to interact with ABCB protein for auxin transport. ABCB could export auxin independently of PIN proteins, but the functional interaction of ABCB-PIN pairs for auxin transport is also considered. Abbreviations are listed in Table 1.

**Table. 1:** List of abbreviations.

ARHGAP	Rho GTPase Activating Protein
BRX	Brevis radix
CaN	Calcineurin
CBF/DREB1	C-repeat binding factor / dehydration-responsive element-binding 1
CCL	Conserved C terminus in the LAZY1 family
CICR	Ca^2+^-induced Ca^2+^-release
D6PK	Serine/threonine-protein kinase
DCC	Disposable cell culture
DEK1	Defective Kernel 1
GNOM	ARF guanine-nucleotide exchange factor
HEK	Human Embryonic Kidney
ISS	International Space Station
LAZY1	Plant-specific genes with unknown molecular functions that are involved in gravitropism
MCA	Mid1 (Yeast mechanosensitive channel) complementing activity protein
MSC	Mesenchymal stem cells
MSL	MscS (bacterial mechanosensitive channel of small conductance) like protein
NFAT	Nuclear factor of activated T cells
NOX	NADPH oxidases
OSCA	reduced hyperosmolality- induced [Ca^2+^]_i_ increase 1
PDZ	Domains bind to a short region of the C-terminus of other specific proteins
PLC	Phospholipase C
RANKL	Receptor activator of nuclear factor-B ligand
RCC	Regulator of chromosome condensation
RCN	Reticulocalbin
RLD	Regulator of chromosome condensation-like domains
ROS	Reactive oxygen species
TRPC	Canonical transient receptor potential channel

Gravisensing apparatuses reside in the endodermal cells of shoots and columella cells of roots, where amyloplast statoliths sediment upon plant reorientation^12^. However, the mechanisms of graviperception remain unsolved. The Cholodny–Went theory explains differential growth in tropisms through the redistribution of the plant hormone auxin in elongating organs^13^. In gravistimulated plants, more auxin accumulates in the lower side than the upper side of shoots and roots in a horizontal position, causing the upward bending of shoots and downward bending of roots. Auxin redistribution following gravistimulation has been verified. Indeed, molecular genetics with many gravitropic mutants has revealed the importance of the roles of auxin transport, redistribution, and response in gravitropism^14^. In particular, the identification of an auxin efflux carrier PIN-FORMED (PIN) was a significant breakthrough in our understanding of the mechanisms for asymmetric auxin transport and distribution in gravistimulated shoots and roots^15,16^. However, unlike some PINs, the pattern of ABCB expression and the phenotypes of ABCB mutants indicate that it is not directly involved in asymmetric redistribution of auxin during gravitropic response^10,11^. In roots, for example, PIN3 and PIN7 localize to the plasma membrane of gravisensing columella cells in the root cap. Within 10 min of gravistimulation by reorienting plant seedlings, PIN3 and PIN7 change their location to the new bottom of the plasma membrane, allowing auxin to move to the bottom side of the root cap. Thereafter, PIN2 localizing to the proximal side of the plasma membrane in the lateral root cap and the epidermis, plays a role in the basipetal transportation of auxin from the bottom side of the root cap to the elongation zone. In gravistimulated roots, auxin redistribution is thus established, and transcriptional regulation depending on the auxin level on the upper and lower sides leads to downward bending. Spaceflight experiments with cucumber seedlings in the Space Shuttle and the International Space Station (ISS) showed that the endodermal cells relocalize an auxin efflux carrier, CsPIN1, because of gravistimualtion in space and laterally transport auxin from the upper to lower flank^17–19^.

Some factors that could be involved in PIN polarization and thereby asymmetric auxin flow have been reported. That is, proteins such as RCN1, PINOID, and D6PK regulate PIN phosphorylation/dephosphorylation^2,20,21^. The phosphorylation status of PIN proteins, together with GNOM-dependent PIN recycling processes, is hypothesized to participate in polar localization of PIN proteins on the plasma membrane. Dynamics of microfilaments and microtubules (MTs) is an important factor involved in the regulation of trafficking of auxin transporters. It is reported that Sorting Nexin 1 (SNX1) plays a role in PIN2 recycling via interaction with MTs-associated protein CLASP^22^.

Recently, it was found that LAZY1 regulates PIN relocalization in gravisensing cells and determines negative gravitropism in shoots and positive gravitropism in roots^23,24^. Interestingly, the alteration of two amino acids in LAZY1 was found to successfully switch negative gravitropism to positive gravitropism in Arabidopsis shoots^25^. Furthermore, it was revealed that upon amyloplast sedimentation, LAZY1/LAZY1-like proteins get polarized to the plasma membrane of the bottom side of gravisensing cells^26^. The conserved C terminus in the LAZY1 family (CCL) domains interact with the Brevis radix (BRX) domains of the regulator of chromosome condensation (RCC)-like domains (RLD) proteins, thereby polarly recruiting RLD from the cytoplasm to the plasma membrane^26^. It was demonstrated that RLD1–4 localize in the root cap and modulate auxin transport through regulation of PIN localization, possibly via a GNOM-like function in PIN trafficking^26^. This process is required for controlling polarized auxin flow and gravitropic response. Thus, amyloplast position itself may play an important role in gravity sensing/signaling as discussed in next section “Gravity sensor in plants.” However, the mechanism underling the LAZY polarization upon amyloplast sedimentation in gravisensing cells still remains unknown.

Auxin biosynthesis and distribution in microgravity were also examined by spaceflight experiments in some plant species. Most of those results showed no differences between space- and ground-grown seedlings. Recently, transformed *Arabidopsis* lines with GFP reporter gene, pDR5r::GFP, pTAA1::TAA1-GFP, pSCR::SCR–GFP and pARR5::GFP, were used for spaceflight experiments on ISS^27^. The expressions of the auxin artificial AuxRE promoter construct (pDR5r::GFP), Tryptophan Aminotransferase of Arabidopsis fusion (pTAA1::TAA1-GFP) and Scarecrow fusion (pSCR::SCR–GFP) were used to monitor auxin level, auxin production and auxin-related signals, respectively. There were no differences in the expression patterns and levels of those genes in the primary root tips of seedlings grown under microgravity and 1 G ground conditions. These results implied that auxin gradient in plants is established independently of gravity. On the other hand, spaceflight experiments with pea and maize seedlings showed altered polar auxin transport in microgravity; polar auxin transport in microgravity was decreased in pea epicotyls and accelerated in maize coleoptiles and mesocotyls compared with the 1 G controls^28,29^. Recent spaceflight experiments immunohistochemically compared PsPIN1 localization in etiolated pea epicotyls grown under microgravity and 1 G conditions in space^30^. PsPIN1 proteins were detected in the lower side of the plasma membrane of 80–90% endodermal cells under artificial 1 G conditions, whereas number of those endodermal cells showing polarized PsPIN1 localization significantly decreased in microgravity. The authors consider the change in PsPIN1 localization pattern as a possible cause for the reduction of polar auxin transport in pea epicotyls under microgravity conditions. In maize seedlings, interestingly, the enhanced accumulation of ZmPIN1and the alteration of ZmPIN1a localization in parenchymatous cells of the coleoptiles were likely responsible for the enhanced polar auxin transport in microgravity^31^. However, species differences of polar auxin transport in microgravity are mysterious.

Thus, the PIN-mediated auxin transport and distribution are essential parts of plant gravimorphogenesis. It should be emphasized that some PIN proteins were verified to be gravity responsive in their relocalization on the plasma membrane of the gravisensing cells by spaceflight experiments. Polarization and function of LAZY1/LAZY1-like proteins appear to play a key role in the gravity-induced PIN relocalization and thereby asymmetric auxin flow. To understand the entire regulatory mechanism of plant gravimorphogenesis, it is important to clarify the graviperception mechanism that leads to the regulation of LAZY1/LAZY1-like proteins polarization and PIN relocalization in gravisensing cells.

### Gravity sensor in plants

As discussed above, plants have a mechanism for gravity sensing using the sedimentation of organelles in order to establish the asymmetric transport of hormones. The most widely accepted model for plant gravity sensing is the starch-statolith hypothesis, in which intracellular sedimentation of the starch-filled organelle (amyloplast) plays a crucial role in the events triggering the initial phases of gravity sensing in plants^32–34^. Recent live-cell imaging technology has revealed, however, that the movement of the amyloplast is not static but saltatory because its dynamics are dependent on both the gravity vector and intracellular environments such as those of the cytoskeleton and vacuole^35^. In the shoot statocytes (endodermal cells) of *Arabidopsis thaliana*, the amyloplasts are tightly surrounded by the vacuolar membrane and are supposed to interact with actin filaments^36^. The abnormal behavior of the vacuolar membrane, however, pushes the amyloplasts to the periphery of the cell in the agravitropic mutant, *shoot gravitropism* (*sgr*) *2*, which restricts the movement of the amyloplasts and renders them nonsedimentable^37^. Therefore, inflorescence stems in *sgr2* mutants do not sense gravity and do not show a gravitropic response because of nonsedimentable amyloplasts^37^. *distorted1* (*dis1*)/*actin-related protein 3* (*arp3*) mutants possess irregular thick actin bundles surrounding amyloplasts in their root statocytes (columella cells), and consequently, the amyloplasts do not sediment fully from the actin filaments, resulting in a reduced gravitropic response in roots^38^. *sgr9* mutants also have nonsedimentable, clustered amyloplasts entangled with actin filaments in the endodermal cells because of an excess of interaction between the amyloplasts and the actin filaments and exhibit a weak gravitropic response^36^. These abnormal phenotypes in both *dis1* and *sgr9* mutants were compensated by disrupting actin filaments, such as through the use of the actin filament-depolarizing drug latrunculin B^36,38^, suggesting that the actin filament is not an essential component for either gravity sensing or gravitropic responses, but rather acts as an intracellular component affecting amyloplast dynamics. Taken together, the dynamics of the vacuolar membrane and actin filaments could diffuse amyloplasts from the bottom of the cell, leading to the nonstatic, saltatory behavior of the plant statolith and amyloplast.

A long-lasting question regarding gravity sensing in plants is how the physical process of amyloplast sedimentation is converted into intracellular signals. The most reasonable models, such as the inner ear (hair cell) system of vertebrates, suggest that amyloplast sedimentation activates mechanosensitive ion channels *via* actin, resulting in intracellular ionic signaling^39–41^. Changes in the gravity vector (inclining the specimens) elevate cytosolic calcium concentrations in *Arabidopsis* seedlings^42,43^. This Ca^2+^ response is attenuated with latrunculin B and mechanosensitive ion channel blockers such as Gd^3+^ ^42^, supporting a model involving actin filaments that function as a tether to activate mechanosensitive channels^39^. Actin filaments may have both a positive role in the activation of mechanosensitive channels upon gravity stimulation and a negative role in the sedimentary dynamics of amyloplasts as discussed above. To demonstrate this, direct observations of Ca^2+^ responses in both the shoot endodermal and root columella cells are needed. An alternative model is the position-sensing hypothesis in which the spatial distribution of amyloplasts upon gravity stimulation is detected as a signal for gravity sensing^44^. In this hypothesis, a putative machinery, rather than mechanosensitive channels sensing the gravitational force exerted on the amyloplasts, detects the position (state) of the amyloplasts in gravity sensing cells, consistent with data indicating that gravitropic responses in wheat coleoptile are dependent on the angle of inclination of the specimens but not on the amplitude of the gravitational force^45^. These data suggest a variety of gravity sensing mechanisms in diverse plant species such as monocots or dicots which are, however, quite different from those of animals.

### Mechanosensitive channels in plants

As discussed above, gravity is the force that generates several effects such as the weight leading to the sedimentation of amyloplast statoliths, deformation and compression of the cells, and fluid shift in vasculature, all of which generate mechanical stresses in plasma and endomembranes. One of the earliest response to changes in gravity vector and magnitude is the Ca^2+^ response, which has been reported in many plant species^46^. Thus, it is plausible that mechanosensitive channels are the primary sensors of plant graviperception evoking the Ca^2+^ response.

MSL proteins share the C-terminal transmembrane (TM) segment corresponding to the pore-forming transmembrane segment of MscS (mechanosensitive channel of small conductance) in *E. coli*^47^. Among the 10 members of the MSL protein in *Arabidopsis*, MSL1 localizes to the inner mitochondrial membrane, whereas MSL2 and 3 are found at the inner plastid membrane, and MSL8, 9, and 10 localize to the plasma membrane^48^. MSL2 and 3 regulate the size and shape of the plastid, and MSL8 is required for the rehydration of the pollen grain, indicating that a major role of MSL proteins is osmotic regulation. Yeast two-hybrid assays demonstrated that MSL2 and 3 interact with each other, suggesting that some MSLs can form heteromeric channels^49^. MCA proteins have been identified as complements of the *mid1* mutant of the yeast that is defective in Ca^2+^ influx^50^. MCA1 and MCA2 localize in the plasma membrane and mediate the cold-induced Ca^2+^ response that leads to cold tolerance according to the CBF/DREB1-independent pathway^51^. MCA1, but not MCA2, is required for the penetration of roots into harder agar and results in the retardation of the leafing and bolting of the mutant. The expression levels of MCA1 and MCA2 were increased under hypergravity conditions in the absence of light, and the hypocotyl elongation under these conditions was attenuated in the overexpressing seedlings. Therefore, MCA proteins might be responsible for resistance to gravity^52^. OSCA proteins, homologous to the TMEM63 family of proteins known throughout eukaryotes, have been identified from *Arabidopsis* mutants exhibiting a low hyperosmolality-induced Ca^2+^ response^53^. An ortholog of animal Piezo protein, the mechanosensitive cation channel for touch sensation and vascular development, is commonly conserved in monocots and suppresses the systemic movement of viruses^54^.

Mechanosensitive cation channel activity in MCAs and OSCAs has been recorded using patch-clamp techniques with heterologous expression in *Xenopus* oocytes and HEK cells^55,56^. Electrophysiological studies using *Arabidopsis* mutants and *Xenopus* oocytes have revealed that MSL proteins show a preference for anions over cations, leading to depolarization of the plasma membrane and the following Ca^2+^ response through the activation of voltage-dependent cation channels^57^. Most recently, a lack of rapidly activated mechanosensitive Ca^2+^-permeable channel activity (RMA) was reported in *Arabidopsis* DEK1 mutants^58^. Although there is no sequence homology, AtDEK1 has a high number of transmembrane helices as with mammalian Piezo proteins, and RMA shows low conductance and rapid inactivation. These electrophysiological studies have demonstrated that the thresholds of membrane stretch for the activation, conductance, and inactivation time constant of plant mechanosensitive channels are varied, even within the same family.

Although series of proteins and their physiological roles have been characterized in numerous aspects, the mechanosensitive channels responsible for gravitropism have not yet been identified. Overlapping tissue expression patterns suggest that mechanosensitive channels in the same tissue share physiological functions. Thus, most mutants, even those that lacking five MSLs (MSL4, 5, 6, 9, and 10), do not show a significant phenotype^47^. Interestingly, most mechanosensitive channels are expressed in the vasculature, where gravity-induced Ca^2+^ response is observed^46^. In root statocytes of *Brassica* grown in ISS, ten-minute onset (µg to 1 g) or removal (1 g to µg) of a gravity-induced Ca^2+^ response in absence of a significant statolith displacement^59^. Changes in gravity vector and magnitude promote a Ca^2+^ response with similar kinetics^46^. It suggests that multiple mechanosensitive channels in plasma- and endomembranes could be differentially activated by gravity, and promotes a small Ca^2+^ response that is amplified by common intracellular machineries. Thus, pharmacological studies suggest that gravity-induced Ca^2+^ response is greatly amplified by Ca^2+^-induced Ca^2+^-release (CICR) from organelles through signaling cascades, including PLC activation^43^. Subcellular and tissue-specific distributions of gravity-induced Ca^2+^ response and the underlying molecular mechanisms should be investigated more deeply in order to understand graviperception mechanisms in plants.

## Gravity sensing in animals

### YAP-mediated gravity response and 3D organ growth and maintenance

Although plants and animals share common mechanisms for gravity sensing, such as the homologous mechanosensitive ion channels discussed above, the transcriptional coactivator Yes-associated protein (YAP) is a mechanosensitive machinery specific to animals. The relationship between gravity and YAP was first revealed by the analysis of the medaka fish YAP mutant. The body of this mutant was flattened because of its inability to withstand gravity^60^. This demonstration that YAP is required for withstanding gravity in generating a 3D body/organ shape first suggested that YAP not only transduces gravity responses as a mechano-transducer^61^, but more strikingly acts as a mechano-effector for withstanding gravity, forming a mechanical negative-feedback^60^. Since YAP is the key regulator orchestrating organ growth^62^, this review will focus on the role of YAP in linking gravity response with organ growth and maintenance.

YAP and its paralog TAZ (transcriptional coactivator with PDZ-binding motif) act as transcriptional co-activators, mainly in the nucleus^63,64^. YAP nuclear localization is controlled mainly by the Hippo pathway and F-actin-mediated signaling responses to diverse signals, e.g., growth factors and mechanical stimuli^65^. YAP is able to expand the organ size when constitutively activated^62^ and is involved in such diseases as cancer and fibrosis^65^.

**Fig. 2:**
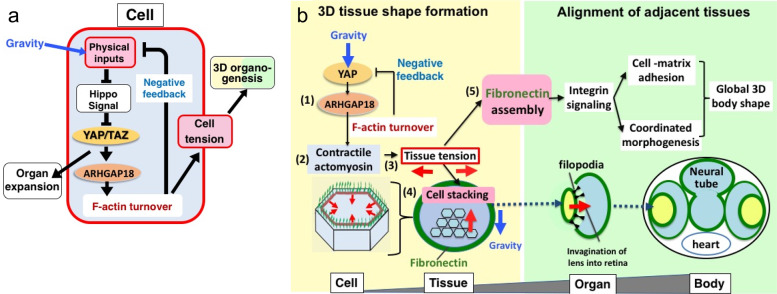
YAP-mediated 3D organ/tissue formation withstanding gravity. **a** Mechanical negative feedback maintaining YAP activity in a cell. **b** YAP-mediated 3D organ/tissue formation withstanding gravity. In (**a**), YAP/TAZ acts as a mechanotransducer and mechanoeffector. As a mechanotransducer, it provides physical inputs, including gravity activation of YAP/TAZ that leads to an expansion of organ size. As a mechanoeffector, it activates YAP, which, in turn, controls F-actin turnover, leading to the suppression of YAP as part of a negative feedback mechanism. F-actin turnover controls the cell/tissue tension that mediates 3D organogenesis. **b** YAP is essential for the formation of complex 3D organs by coordinating 3D tissue shape (left) and tissue alignment (right). In response to external forces, including gravity, YAP activates (1) ARHGAP18 expression, which mediates (2) contractile actomyosin formation controlling (3) tissue tension. Tissue tension is required for both (4) cell stacking to form a 3D tissue shape and (5) fibronectin assembly required for adjacent tissue alignment, e.g., the alignment of the lens and eye-cup.

The discovery that YAP could act as a mechanoeffector uncovered a negative feedback control of YAP activity: F-actin polymerization activates YAP^66^ and its target gene ARHGAP18 and then negatively regulates F-actin polymerization, suppressing YAP activity^60^ (Fig. 2a). This is a mechanical negative feedback since the negative regulation of F-actin polymerization by YAP optimizes F-actin turnover and maximizes actomyosin contractility, i.e., cell/tissue tension. Cell/tissue tension then controls 3D tissue formation and tissue alignment necessary for generating a 3D organ consisting of multiple tissues, e.g., an eye consisting of the lens and eye cup^60^ (Fig. 2b). It is hypothesized that a YAP-mediated response to gravity is involved in the maintenance of bones and skeletal muscles, since YAP is known to control the organ size through Hippo signaling and is expressed in the stem cells of many organs, including skeletal muscles.

YAP orchestrates the response to gravity by controlling actomyosin contractility by negatively regulating F-actin polymerization through its target gene ARHGAP18. Since actomyosin both generates mechanical forces and acts as a mechanical sensor^67^, actomyosin is a putative gravity sensor. Gravity could promote F-actin polymerization, activating YAP in order to maintain a 3D organ size. This is consistent with reports that simulated microgravity inhibits the osteogenic differentiation of mesenchymal stem cells via the de-polymerization of F-actin that inhibits TAZ nuclear translocation^68^. Further detailed studies are necessary to elucidate the mechanisms by which the YAP-mediated gravity response is linked with organ growth and maintenance. These studies will be useful in alleviating compromises to health, such as the loss of bones and skeletal muscles that arises from periods of “life in space.”

### Gravity sensing in bones

**Fig. 3:**
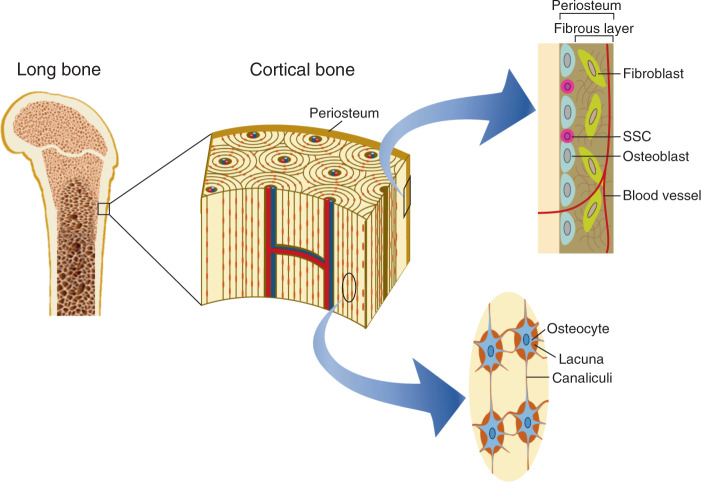
The structure of the long bone, cortical bone, and periosteum. Osteocytes are embedded in the lacuna of the bone matrix and are connected with each other through dendrites surrounded by canaliculi. At the periphery of the bone, SSCs, and fibroblasts form the periosteum together with osteoblasts. Osteocytes and the periosteum are mechanical sensors in the bone tissue.

Bone loss is one of the major health problems facing organisms that experience life in space. The structure of bones is shown in Fig. 3. Here we discuss the sensing mechanisms of gravitational loads in the trabecular bone and cortical bone.

### Mechanical sensing by osteocytes, a commander for osteoclasts and osteoblasts

Bone homeostasis is maintained as a result of the balanced action of osteoblasts for bone formation and osteoclasts for bone resorption^69^. In bone destructive diseases, such as osteoporosis, bone resorption is favored over bone formation, leading to bone loss. In particular, bone loss in unloading conditions, such as microgravity in space, is caused by enhanced bone resorption by osteoclasts and suppressed bone formation by osteoblasts.

Osteocytes, another type of bone cell, are differentiated from osteoblasts and embedded in the bone matrix have many dendrites formed through osteocytogenesis that communicate either with each other or with the osteoblasts and osteoclasts at the bone surface^70^. Osteocytes also have a critical role in bone homeostasis, functioning as a commander for osteoclasts and osteoblasts by regulating the expression of genes involved in the receptor activator of nuclear factor-B ligand (RANKL), an osteoclast differentiation factor, and as a negative regulator of osteoblast differentiation by sclerostin (Sost)^71^. Importantly, expressions of these genes vary in response to mechanical loading or unloading to osteocytes in the bone. Osteocyte cell body and dendrites reside in the lacunae and canaliculi, respectively, and mechanical loading induces minute changes in the structure of the bone that generate interstitial fluid flow in the lacuno-canalicular system. This flow acts as a mechanical loading, similar as pressure and shear stress, and affects osteocytes directly^72^.

Such mechanical loadings to osteocytes as described above can activate mechanotransduction mediators such as ion channels, connexins, integrins, and cytoskeleton-related molecules^73^. In addition, the cytosolic signaling adapter protein p130Cas, a cellular mechanosensing molecule^74^, is involved in the regulation of bone homeostasis in response to mechanical loading in osteocytes^75^. Interestingly, p130Cas translocates into the nucleus and negatively regulates NF-κB activity to suppress bone resorption by downregulating the expression of RANKL. These findings suggest that the p130Cas- NF-κB axis in osteocytes is a potential target for treatment against disuse osteoporosis.

Although the critical significance of mechanical loading to the bone has been clearly elucidated, a large portion of the molecular mechanisms underlying the mechanical regulation of bone homeostasis is not understood. Efforts to clarify these mechanisms will be a promising strategy to prevent bone loss during future space missions.

### Periosteum might sense physical loading

The deterioration of the bone microarchitecture during spaceflight occurs not only in the trabecular bone but also in the cortical bone^76^. It is thought to be triggered by enhanced osteoclast-mediated bone resorption at the endocortical surface and suppressed bone-forming activity in the periosteum. The periosteum, the highly vascularized outer membrane that covers all bones except for joints, generates the cortical bone in physiological and pathological situations through the provision of osteoblasts^77^. The periosteum contains two layers: an outer layer of fibroblasts and an inner layer composed of bone-forming osteoblasts. Although the periosteum is not highly sensitive to mechanical loading compared to the endocortical surface, it nonetheless responds to loading and gives rise to bones in a variety of animal models. The unloading model of the hind limb reduces bone formation in the cortical bone as well as in the trabecular bone^78^. Conversely, periosteal bone formation is stimulated by enhanced loading using in vivo models of axial loading and three-point bending^79,80^. The alteration of gene expression patterns and cell morphologies within the periosteum after loading provides evidence that periosteal cells sense loading stimuli^80,81^.

### Mechanical loading is possibly translated into bone formation through the periosteal skeletal stem cell

Among the cells responsible for sensing physical loading is skeletal stem cell (SSC) because loading-induced bone formation requires activation of the periosteal SSC to give rise to osteoblasts. The periosteal SSC displays a unique gene expression pattern and exhibits high regenerative capacity in response to bone injury when compared to bone marrow skeletal stem cells (BMSC)^82^. A recent study has revealed that Cathepsin K (CTSK)-lineage populations within the periosteum contain postnatal self-renewing and multipotent stem cells^83^. The deletion of Osterix, encoded by the *Sp7* gene, in CTSK-lineage cells results in impaired bone formation and fracture healing^83^. Furthermore, Prx1-lineage mesenchymal cells that contain SSC sense loading stimuli through their primary cilia indicate loading-dependent bone formation^78,82^. More recently, Nestin^+^ and Leptin^+^ cells have been shown to generate osteoblasts for periosteal bone formation^84^. On the other hand, some studies have shown that loading alters gene expression patterns, including extracellular molecules in osteoblasts^85^. Thus, the osteoblast may function as a mechanotransducer that induces osteogenic differentiation of SSCs in the periosteum. Accordingly, direct or indirect loading can activate several types of SSC to induce cortical bone formation.

### Gravity sensing in muscles

Muscle atrophy is another major health problem in life in space and involves the decrease of muscle mass in response to the reduction of hemodynamic loads. It is known to be caused by microgravity, long term bed rest, and cancer cachexia^86^. Unloading-induced muscle wasting is mediated by a decrease of protein synthesis in the homeostasis of muscle cells and an increase of catabolism. Consequently, there must be a molecule that senses and transduces the signals originating from mechanical loading. One of the candidates for such a load transducer is a nonselective cation channel, the canonical transient receptor potential channel (TRPC). Members of the TRPC channels, namely, TRPC1, TRPC3, and TRPC6, are reportedly activated downstream of mechanical signals in addition to phospholipase C-coupled cell surface receptor activation^87^. TRPC channels play important roles in the activation of protein phosphatase calcineurin (CaN). CaN regulates the Ca^2+^-dependent transcription factor, the nuclear factor of activated T cells (NFAT), and the peroxisome proliferator-activated receptor γ^88^. Both proteins are important for myogenesis. Exposure of C2C12 skeletal myoblasts to microgravity induces the reduction of TRPC1 expression, which arrests the cell cycle at the G2/M phase, thereby inhibiting myoblast proliferation^89^. The importance of TRPC1 has also been demonstrated in muscle regrowth after unloading-induced atrophy. Hind limb unloading induces the reduction of TRPC1 expression, which persists even after reloading^90^. The expression of the TRPC3 channel is also suppressed at complete atrophy and in the early recovered phase^90^. These changes in the expression of the TRPC1 and TRPC3 channels are consistent with the muscle mass, suggesting that these channels play important roles in load-dependent muscle growth.

It is widely accepted that oxidative stresses caused by the aberrant production of reactive oxygen species (ROS) or reactive nitrogen species are key regulators for catabolic muscle wasting^91^. ROS are produced as a byproduct of the mitochondrial respiratory chain or are produced enzymatically by NADPH oxidases (NOX) within the cell. In cardiac muscles, ROS production by the NOX2 protein is physiologically important for Ca^2+^ homeostasis and is activated mechanically during diastole^92^. However, in pathological conditions, NOX2-mediated ROS production causes cardiac remodeling in response to various stresses. It has also been noted that pathological situations in muscle tissue can engender abnormal Ca^2+^ signaling. Since some NOX isoforms require Ca^2+^ for activation, it is plausible that there exists a crosstalk between pathological NOX activation and abnormal Ca^2+^ signaling. TRPC3 and NOX2 proteins exist at this crossroads of signaling pathways. Additionally, it has been demonstrated that the TRPC3 channels play an important role in NOX2 protein stabilization by protecting them from proteasomal degradation^93^. ROS production mediated by TRPC3 and NOX2 coupling causes cardiac muscle atrophy in stressed hearts, in which the hemodynamic load is reduced^94,95^. Therefore, TRPC channels might have dual roles in unloading-induced muscle atrophy: the first is the regulation of myoblast proliferation *via* CaN activation, and the second is the production of ROS, which induces catabolic remodeling of muscle tissue.

### Gravity sensing in mesenchymal stem cells

Mesenchymal stem cells (MSCs) are crucial in the field of regenerative medicine by virtue of their self-renewal and multi-differentiation potentials^96^. MSC self-renewal and differentiation are known to be controlled by a diverse set of soluble factors, including growth factors or cytokines. In addition, the fate of MSCs has been shown to be influenced by mechanical stresses or surrounding physical microenvironments, such as substrate stiffness^97^, or changes in gravity. Many space experiments and ground-based studies have demonstrated that MSCs are very sensitive to the modulation of gravitational stimuli and exhibit various responses against such effects^98^. The exposure of MSCs to microgravity or simulated microgravity induces characteristic physiological responses, including remodeling of the cytoskeleton and the disruption of the stress fiber^99,100^, reduced activity in transcriptional coactivator YAP/TAZ^68^, suppression of osteoblastic differentiation, and the promotion of adipogenesis^101,102^, some of which were also observed in other nonspecialized animal cells.

**Fig. 4:**
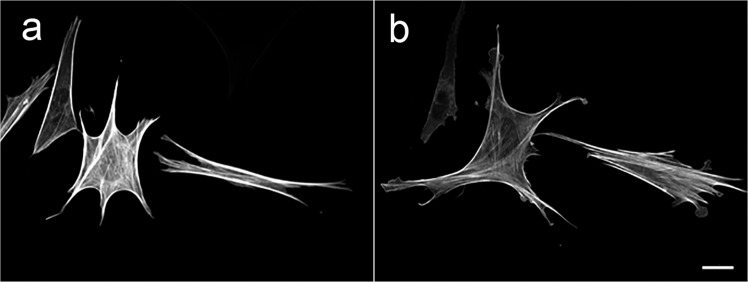
Stress fiber remodeling in MSCs exposed to simulated microgravity as analyzed by confocal fluorescence microscopy. MSCs expressing Lifeact-TagGFP2 contained thick stress fibers under 1 G conditions (**a**), whereas an exposure to simulated microgravity for 6 h led to the appearance of thinner stress fibers (**b**). The images in (**a**) and (**b**) showing the same field of view, were recorded, processed and presented in an identical condition (Kobayashi, unpublished). Scale bar: 20 μm.

How can MSCs sense a microgravity environment? Ordinary mechanical forces, including stretch or shear stress, can be sensed by animal cells through cell mechanosensors that convert mechanical stimuli into electrical or chemical signals. To date, mechanosensitive channels, focal adhesion proteins (p130Cas and Talin), and actin fibers have been established to function as mechanosensors for various types of cells. It has been postulated that MSCs also utilize these common sensors to detect changes in gravity, since MSCs have no specific gravity sensors, as is the case for organs such as the animal gravity sensor statocyst. Recent studies have proposed that the cytoskeleton may function as an initial sensor for microgravity^103^. In the early phase (30 min to 6 h) of exposure to microgravity, environmental changes experienced by the cytoskeleton have been observed, including a reduced amount or thinning of stress fibers^103^ (unpublished data in Fig. 4) and the redistribution of microtubules. In addition, genetic restoration of the arrangement of actin fibers or the pharmacological stabilization of actin cytoskeleton could maintain the osteogenic differentiation of MSCs under modeled microgravity^68,99^. This indicates that changes in the actin cytoskeleton in the cells transferred under microgravity conditions could have a crucial role in cellular responses against changed gravity. However, it remains unclear if the cytoskeleton acts as an initial and primary mechanosensor for gravity sensing^103^. It has been proposed that the loss of gravitational forces acting on heavy organelles, including the nucleus and mitochondria, could affect the cytoskeleton. Further studies will provide deeper insight regarding gravity sensing and transduction.

### Gravity sensitivity of the cell cycle

Regulation of the cell cycle is crucial for the maintenance of organs, such as bones and muscles. Technological innovation in bio-imaging has recently used fluorescent proteins, and we advocate real-time, single-cell imaging techniques to accurately and comprehensively dissect molecular and cellular principles and explain natural and sample-originated heterogeneity in biology^104^. For example, our fluorescent, ubiquitination-based cell cycle indicator (Fucci) technology harnesses the cell-cycle-dependent proteolysis of Cdt1 and Geminin fused to fluorescent proteins of different colors^105,106^. Although considerable progress has been made toward understanding the mechanisms of cell cycle progression on Earth^107^, much less is known about how cell proliferation is affected by microgravity in outer space, where humans will live in the future.

As discussed above, regulation of the cell cycle is crucial for growth and maintenance of organs. To directly understand the precise molecular mechanisms of how individual cells in organs respond to microgravity, several simulated microgravity experiments have been performed on the ground using cultured cells. The relationship between gravity force and cell cycle progression has been reviewed^108^, but remains controversial. Because individual studies use different experimental setups and different types of cells, discrepancies in results might arise inevitably. The 3D-clinorotation system provides time-averaged simulated microgravity as an alternative to real microgravity conditions^109^, and the effects of microgravity on living cells have been studied using this device. For example, cultured cells were exposed to microgravity for several days. Cells were seeded in 25 cm^2^ flasks or on a DCC (disposable cell culture) plate^110^, which were then fully filled with a CO_2_-equilibrated medium and accommodated on the 3D-clinorotation device. After exposure, the number of cells was quantified to determine whether microgravity inhibits the proliferation of cells.

To visualize the progression of the cell cycle of cultured cells in real time at the single-cell level during exposure to microgravity, we are currently developing a 3D-clinorotation 2D-microscopy system. The system accommodates a portable fluorescence microscope that we invented on the basis of a smartphone and a DCC-G (glass-based DCC^110^) plate for fluorescence observation. This system can be used in the cell biology experiment facility of the ISS. We have already generated a variety of human cell lines that constitutively express Fucci probes. Hopefully, this will enable us to better understand how cells behave in outer space.

## Conclusion

Response to gravity is a cellular process of mechanotransduction in both plants and animals. Interestingly, although plants and animals seem to be very genetically distant, they share common mechanisms for gravity sensing, e.g., an actin cytoskeleton and mechanosensitive ion channels combined to this skeleton. On the other hand, animals evolved unique systems for gravisensing as exemplified by the transcriptional coactivator YAP/TAZ, which affects the cell fate of bones, muscles, and stem cells. Knowledge derived from extensive studies of gravisensing will contribute to medicine on Earth, e.g., understanding osteoporosis, muscle atrophy, and cancer biology. However, in the present age of preparation for human space exploration and colonization, or example through the Moon base and Mars exploration, understanding cellular responses to gravity will form the foundations of living in space.

## Data Availability

All data are available in the main text.
